# Contrasting spatial patterns and ecological attributes of soil bacterial and archaeal taxa across a landscape

**DOI:** 10.1002/mbo3.256

**Published:** 2015-04-28

**Authors:** Florentin Constancias, Nicolas P A Saby, Sébastien Terrat, Samuel Dequiedt, Wallid Horrigue, Virginie Nowak, Jean-Philippe Guillemin, Luc Biju-Duval, Nicolas Chemidlin Prévost-Bouré, Lionel Ranjard

**Affiliations:** 1 INRA, UMR1347 AgroécologieBP 86510, F-21000, Dijon, France; 2 INRA, US1106 InfoSolF-45075, Orléans, France; 3 INRA, UMR1347 Agroécologie-Plateforme GenoSolBP 86510, F-21000, Dijon, France; 4 AgroSup Dijon, UMR1347 AgroécologieBP 86510, F-21000, Dijon, France

**Keywords:** agroecology, bacterial communities, ecological attributes, landscape, soil, spatial distribution

## Abstract

Even though recent studies have clarified the influence and hierarchy of environmental filters on bacterial community structure, those constraining bacterial populations variations remain unclear. In consequence, our ability to understand to ecological attributes of soil bacteria and to predict microbial community response to environmental stress is therefore limited. Here, we characterized the bacterial community composition and the various bacterial taxonomic groups constituting the community across an agricultural landscape of 12 km^2^, by using a 215 × 215 m systematic grid representing 278 sites to precisely decipher their spatial distribution and drivers at this scale. The bacterial and Archaeal community composition was characterized by applying 16S rRNA gene pyrosequencing directly to soil DNA from samples. Geostatistics tools were used to reveal the heterogeneous distribution of bacterial composition at this scale. Soil physical parameters and land management explained a significant amount of variation, suggesting that environmental selection is the major process shaping bacterial composition. All taxa systematically displayed also a heterogeneous and particular distribution patterns. Different relative influences of soil characteristics, land use and space were observed, depending on the taxa, implying that selection and spatial processes might be differentially but not exclusively involved for each bacterial phylum. Soil pH was a major factor determining the distribution of most of the bacterial taxa and especially the most important factor explaining the spatial patterns of *α*-Proteobacteria and Planctomycetes. Soil texture, organic carbon content and quality were more specific to a few number of taxa (e.g., *β*-Proteobacteria and Chlorobi). Land management also influenced the distribution of bacterial taxa across the landscape and revealed different type of response to cropping intensity (positive, negative, neutral or hump-backed relationships) according to phyla. Altogether, this study provided valuable clues about the ecological behavior of soil bacterial and archaeal taxa at an agricultural landscape scale and could be useful for developing sustainable strategies of land management.

## Introduction

Spatial patterns, based on describing the distribution of living organisms in relation to space and environmental heterogeneity, provide a key to understanding the structure and function of soil biodiversity (Martiny et al. 2006). In contrast to macro-organisms, the description of spatial patterns of soil microorganisms is recent, but has gained attention due to their key role in ecosystem services (Maron et al. 2011). Soil microbial communities are known to exhibit heterogeneous and structured spatial patterns at various scales ranging from the microscale (soil aggregates, *μ*m) to global scale (continent, >100,000 km^2^) (Nunan et al. 2003; Dequiedt et al. 2009; Franklin and Mills 2009; Lauber et al. 2009; Griffiths et al. 2011). A large body of proof has emerged from these patterns suggesting that the abundance, diversity and assemblages of soil microbial communities are mainly determined by soil properties, plant-cover and land management, rather than by geographical barriers or climate. Thus, environmental selection (aka, niche-based process) by proximal factors would be the main process that shapes soil microbial diversity (Drenovsky et al. 2010; Ranjard et al. 2013). However, other processes based on the neutral theory have recently been shown to successfully predict nonrandom patterns of distribution (Hubbell 2001), and indicate that dispersal limitation could also significantly contribute in shaping patterns of microbial communities (Bell 2010; Stegen et al. 2012).

Although the processes and drivers shaping the bacterial community assembly as a whole have now been deciphered, those influencing the various bacterial taxonomic groups which constitute the community are still little known or understood. This has resulted in a lack of knowledge concerning the ecological attributes of soil indigenous bacterial taxa, which in turn limits our understanding and ability to predict community composition according to surrounding environmental conditions (Fierer et al. 2007; Maron et al. 2011) as well as our ability to link microbial diversity with soil functioning (Maron et al. 2011). Regarding applied ecology, this absence of knowledge is hampering the development of sustainable ecosystem management strategies based on soil microbiological resources (Levin 1992).

Spatial ecology, based on applying meta-analysis approaches under a wide range of environmental conditions, is providing useful information about the ecological attributes of indigenous soil bacterial taxa. Various authors have used spatially explicit approaches to show that the relative abundances of several bacterial taxa display contrasting patterns, thereby highlighting their distinct ecological attributes and confirming the ecological coherence of bacterial taxonomy (Philippot et al. 2009; King et al. 2010). Fierer et al. (2007) used meta-analysis approaches to differentiate soil bacterial taxa into ecologically meaningful categories based on the *r-*/*K-*selection continuum, with *r-*strategists maximizing their intrinsic rate of growth when resources are abundant while *K-*strategists are better adapted to compete and survive when resources are limited (Pianka 1970). However, all these studies were conducted on contrasting (a priori) environmental gradients, and did not provide precise insights into the role and ecology of bacterial taxa or of the complexity of the potential ecological niches occupied by bacteria. To be able to draw conclusions about the different processes involved in community assembly, it is now crucial to decipher more precisely and with greater genericity the ecological attributes of soil microbial taxa by studying their distribution at different scales and integrating the different environmental parameters involved such as soil types, land use, climate, geomorphology, and space.

In a previous study (Constancias et al. under revision), the distribution of microbial abundance and bacterial community diversity (richness, evenness and Shannon's index) was investigated across a landscape of 12 km^2^, offering an opportunity to decrypt the relative influence of soil properties and land management in shaping soil bacterial communities. The landscape, as compared to larger scales, was characterized by a smaller variability in soil properties and also by a mosaic of different types of land use constituted by forest and agricultural plots with contrasting cropping intensity. Soils (*n* = 278) were sampled within a systematic sampling grid covering the entire landscape. Soil physicochemical properties and land management characteristics were determined for each sample. Bacterial diversity was characterized by massive inventory of the 16S rRNA gene sequences amplified from soil DNA. In addition to demonstrating the heterogeneous and spatially structured distribution of microbial abundance and diversity across the landscape, variance partitioning revealed that bacterial richness is mainly driven by soil texture and pH whereas land management is a strong determinant of microbial abundance and bacterial evenness. Altogether this previous study demonstrated the relevance of the landscape scale for deciphering microbial distribution patterns and processes, and for evaluating the effects of land management strategies on soil microbial resources.

In the present study we focused on the distribution of the composition of bacterial and Archaeal communities and of the various taxonomic groups constituting the community across the landscape. The following questions were addressed: are all taxa heterogeneously distributed at this spatial scale? Do they exhibit the same patterns? Which drivers or ecological attributes characterize each bacterial and archeal taxon at this scale? To answer these questions, a geostatistical approach was used to map and describe the spatial variability of community structure and taxa, and a variance partitioning approach was applied to identify and rank the ecological attributes for each taxon. Spatial descriptors were also integrated into the analysis to better interpret their relative contributions to taxa variation across a landscape and to examine other neutral processes shaping bacterial and archeal taxa distribution.

## Materials and Methods

### Site, sampling strategy, and data collection

The study was carried out on a monitored landscape of 13 km² located in Burgundy, France (Fénay, Lat: 47°14′37″N, Long: 5°03′36″E) characterized by deciduous oak-hornbeam forests (3.86 km^2^) and intensive agricultural croplands (9.22 km^2^) mainly under winter crops (winter wheat, oilseed rape) in rotation with late-sown crops (spring barley). The whole area is flat or slightly sloping, under a continental climate with a mean annual air temperature of 10.4°C and a mean annual rainfall of 762 mm (period 1968–2011). Land management practices were clustered into six categories (from forest to agricultural plots with a gradient of cropping intensity).

The sampling design, based on a square grid with spacing intervals of 215 m, covered the entire landscape and corresponded to 248 sites. It also included 30 additional sites positioned within the grid for exploring the variation over distances less than 215 m. All sites were sampled in September 2011. At each of the 278 sampling locations, five soil cores (core diameter: 5 cm; 0–20 cm depth) were randomly collected from a 4 m^2^ area in the inter-row for agricultural sites and at least 1 m away from trees, then bulked, and 2 mm-sieved before being lyophilized at −80°C and finally archived at −40°C.

Samples were randomized before physicochemical and bacterial community characterizations to avoid any batch effect. Analyses of physicochemical properties (pH, organic carbon, total nitrogen, CaCO_3_, clay, silt and sand) were carried out by the Laboratoire d'analyse des sols d'Arras of INRA (http://www.lille.inra.fr/las) as described in Dequiedt et al. (2009).

### Pyrosequencing of 16S rRNA gene sequences

Soil microbial DNA was extracted using the GnsGII procedure developed by Plassart et al. (2012). Crude DNA was then purified using a MinElute PCR purification kit (Qiagen, Courtaboeuf, France) and quantified using the QuantiFluor staining kit (Promega, Madison, USA), prior to further investigations.

The 16S rRNA V3-V4 gene region was targeted for amplification, using primers F479 (5′-CAGCMGCYGCNGTAANAC-3′) and R888 (5′-CCGYCAATTCMTTTRAGT-3′) in a nested PCR strategy to add the 10-bp multiplex identifier (MID) barcode as initially described by Plassart et al. (2012). Equal amounts of each sample were pooled, and all further steps (adapter ligation, emPCR and 454-pyrosequencing) were carried out by Beckman Coulter Genomics (Danvers, MA) on a 454 GS-FLX-Titanium sequencer (Roche Applied Science, Indianapolis, Indiana).

The raw data sets are publicly available in the EBI database system (in the Short Read Archive) under project accession no. PRJEB5219.

### Bioinformatics analysis

The generated sequences were subjected to bioinformatic analysis using the GnS-PIPE developed by the GenoSol platform (INRA, Dijon, France) and initially described by Terrat et al. (2012). After an initial quality filtering step (>350 bp, no base ambiguity), reads were aligned with infernal alignments that use the secondary structure of the 16S rRNA gene (Cole et al. 2009) and clustered at 95% sequence similarity into operational taxonomic units (OTU). This clustering step was done using a custom PERL program that does not consider differences in homopolymer lengths, which can cause the main 454-pyrosequencing errors (Balzer et al. 2011). Each sample was then randomly rarefied at a sequencing depth of 10,800 quality sequences to allow rigorous comparison of the data. Community structure was characterized using weighted UniFrac distance (Lozupone and Knight 2005) calculated with the PycoGent package (Knight et al. 2007) on a phylogenetic tree computed using FastTree and the most abundant sequence to represent each OTU. Quality reads were used for taxonomy-based analysis by similarity approaches using USEARCH (Edgar 2010) against the corresponding Silva database (Quast et al. 2013).

### Metadata analysis

#### Environmental variability of the studied landscape

The variability of soil physicochemical properties across the studied area was assessed by subjecting the data to principal component analysis (PCA). Land management practices over the entire landscape were summarized by performing a factor analysis for mixed data to define land management clusters using the *FactoMineR* package (Lê et al. 2008) with land use, soil tillage, crop rotation diversity (number of plant types in the crop rotation), and the pesticide treatment frequency index, as data input. These clusters followed a gradient in cropping intensity based on soil disturbance and in the diversity and persistence of plant cover that is, Forest (forest, no-tillage, no catch-crop, *n* = 44); Perennial plant cover (three frequently mown) grasslands, three blackcurrant (*Ribes nigrum*) and one Miscanthus (*Miscanthus giganteus*), *n* = 7); Catch Crop (agricultural plot, minimum tillage, catch-crop, *n* = 22); Minimum tillage (agricultural plot, minimum tillage, no catch-crop, *n* = 57); Mechanical hoeing (agricultural plot, mechanical hoeing, no catch-crop, *n* = 33) and Conventional tillage (agricultural plot, conventional tillage, no catch-crop, *n* = 104).

#### Ordination of microbial community structure

Differences in community structure between samples were visualized by applying the weighted UniFrac metric and Nonmetric multi-dimensional scaling (NMDS). Soil physicochemical parameters and the relative abundance of the most dominant bacterial and archeal phyla and Proteobacteria classes were incorporated into the analysis by vector fitting against the bi-plot ordination of community structure. Significance of the vectors was assayed by 999 permutations. Only the most significant (*P *<* *0.001) vectors harboring a correlation ≥0.20 relative to the two NMDS axes were represented.

#### Interpolated mapping

A geostatistical method was used to map soil physicochemical properties (i.e., sample scores on the first three principal components of the PC Analysis conducted on physicochemical characteristics), microbial community structure (i.e., sample scores on the two axes of the NMDS analysis run on the weighted UniFrac distance matrix) and the relative abundance of the most discriminant bacterial and archeal phyla and Proteobacteria classes. As these variables did not follow the strictly required Gaussian distribution, they were first transformed using the nonparametric rank-order (or normal scores) transformation prior to considering the spatial correlations (Juang et al. 2001). It is usual, in geostatistical analysis, to compute an estimate of a variogram model based on the observations which describe the spatial variation of the property of interest. This model is then used to predict the property at unsampled locations using kriging (Webster and Oliver 2007). A common requirement for variogram estimation is first to calculate the empirical (so-called experimental) variogram by the method of moments (Matheron 1965), and then to fit a model to the empirical variogram by (weighted) nonlinear least-squares. We also investigated an alternative method which uses maximum likelihood to estimate the parameters of the model directly from the data, on the assumption that this displays a multivariate normal distribution. We selected the Matérn model which can simultaneously describe several spatial processes (Minasny and McBratney 2005). The validity of the fitted geostatistical model was assessed in terms of the standardized squared prediction errors method (SSPE) using the results of a leave-one-out cross-validation. If the fitted model provides a valid representation of the spatial variation of the soil or microbial property, then these errors display a *χ*^2^ distribution which has a mean of 1 and median 0.455 (Lark 2002). The mean and median values of the SSPE were also calculated for 1000 simulations of the fitted model to determine the 95% confidence limits. An ordinary kriging estimation was performed in the standardized-rank space and the kriging estimates were then back-transformed into the original space. The geostatistical analysis gstat and GeoR R package for variograms analysis and kriging were used (Ribiero and Diggle 2001).

#### Variance partitioning of community dissimilarity and of the relative abundance of bacterial and archaeal taxa

Partial regression models were conducted to estimate the contribution of physicochemical parameters, land management and space in determining variation in community dissimilarity as well as the spatial distribution of bacterial and archaeal taxa. Among the eight measured physicochemical properties, silt was removed because of co-linearity with sand and clay, and nitrogen content because of its correlation with organic content (*r* = 0.92, *P *<* *0.001). In addition to the six retained physicochemical properties and the clusters summarizing land management intensity, space was characterized by using a Principal Coordinates of a Neighbour Matrix approach (PCNM). The PCNM method was applied to the geographic coordinates and yielded 76 PCNM, representing the multiple spatial scales that the sampling scheme could perceive (Ramette and Tiedje 2007). Quantitative response and explanatory data were, respectively, log-transformed and standardized to provide an approximated Gaussian and homoscedastic residual distribution. For each taxon, physicochemical and land management variables were selected by multiple regression analysis using a stepwise selection procedure, which maximized the adjusted R² (in order to maximize the explained variation by the model) and minimized the Akaike Information Criterion (AIC, in order to discard previously retained variables that reduced the overall predictive power). Spatial descriptors were then selected from the model residuals, in order to strictly identify the spatial autocorrelation that did not correspond to spatially structured environmental variables. These selection steps enabled us to exclude those variables that did not contribute significantly to the explained variation (*P *<* *0.001), thereby limiting overfitting and problems due to co-linear variables (Ramette 2007). The respective effects of each explanatory variable, or combinations thereof, were determined by (1) partial regression for the relative abundance of taxa and (2) distance-based redundancy analysis (db-RDA, Ramette and Tiedje 2007; Bru et al. 2010). The statistical significance was assessed by 999 permutations of the reduced model. All these analyses were performed with R (http://www.r-project.org/) using the vegan package (Oksanen et al. 2011).

## Results

### Landscape heterogeneity of environmental parameters

**Figure 1:**
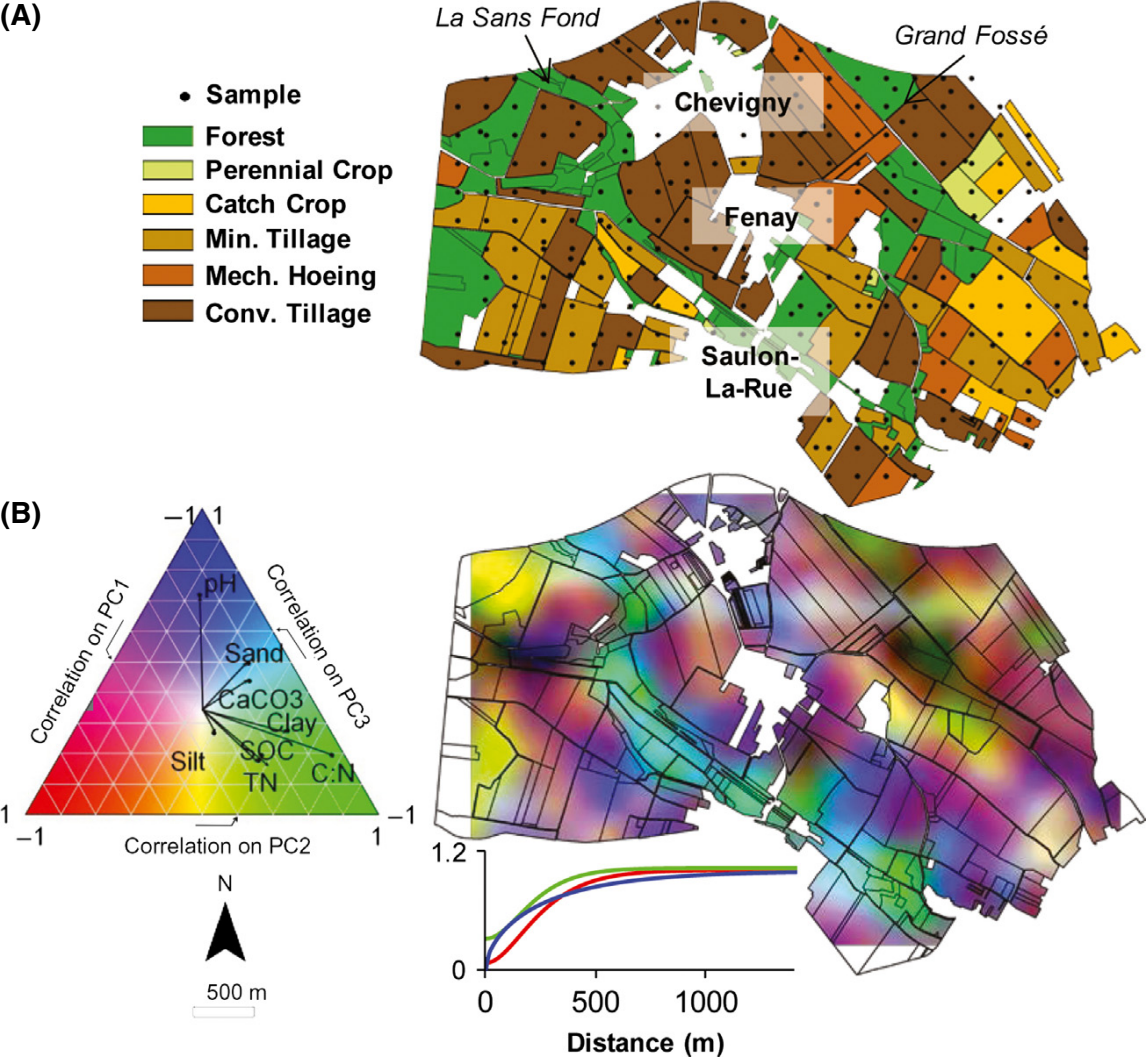
Maps of environmental characteristics of the Fénay landscape. (A) Maps of land management clusters including the samples location, the two rivers and the local villages in the studied area. (B) Maps of samples scores on the three-first axes of the principal component analysis conducted on the physicochemical data set: red green blue RGB color chart, Principal Component1: red, PC2: green, PC3: blue. This approach summarizes the physicochemical properties of the studied area on a single map. Correlations between axes and variables are represented to the right of the map in a triangular diagram to match the color chart. Matérn model semi-variograms of the related PC axis used to produce robust kriging are provided beside the map.

The studied landscape was characterized by alkaline fine-textured soils with a mosaic of different types of land management constituted by forest (18% of the area) and agricultural plots (82% of the area, Fig.1A) subjected to contrasting agricultural practices. Land management was clustered into six categories to depict land management intensity (from forest to agricultural plots with a gradient of land management intensity – see Materials and Methods and Fig.1A).

Most of the soils were silty (median 56.7%) or clayey (median 34.3%) with alkaline pH (median 8.0, Table S1). Organic carbon and total nitrogen contents were highly correlated (*r* = 0.92, *P *<* *0.001) and ranged from 1.74 to 174 and 0.835 to 14.6 g · kg^−1^, respectively (Table S1). Soil properties were spatially structured in patches ranging from 600 to 900 m (Table S2), which reflected both the distribution of land management categories and the variations in pedological patterns (Fig.1A and B). Due to the local chalky limestone characteristics, all soils located along the “Sans Fond” riverbed exhibited similar specific features (higher organic carbon, nitrogen and CaCO_3_ contents, coarser texture and higher pH, Fig.1) whatever the type of land management. On the other hand, samples under forest land management located at the West of the studied area and along the “*Grand Fossé*” riverbed exhibited significant lower pH and higher organic carbon and nitrogen contents and C:N ratio (*P *<* *0.05 in all cases, Fig.1A, yellow patches B). Agricultural plots in the conventional-tillage and mechanical hoeing clusters were mainly situated between the villages of “Chevigny” and “Fénay” whereas most plots in the minimum tillage cluster (with or without catch crop) were found to the extreme south-west and south-east. The forests plots were mainly situated beside the two rivers (“La Sans Fond” and “Grand Fossé,” Fig.1A).

### Microbial composition variation and mapping across landscape

**Figure 2:**
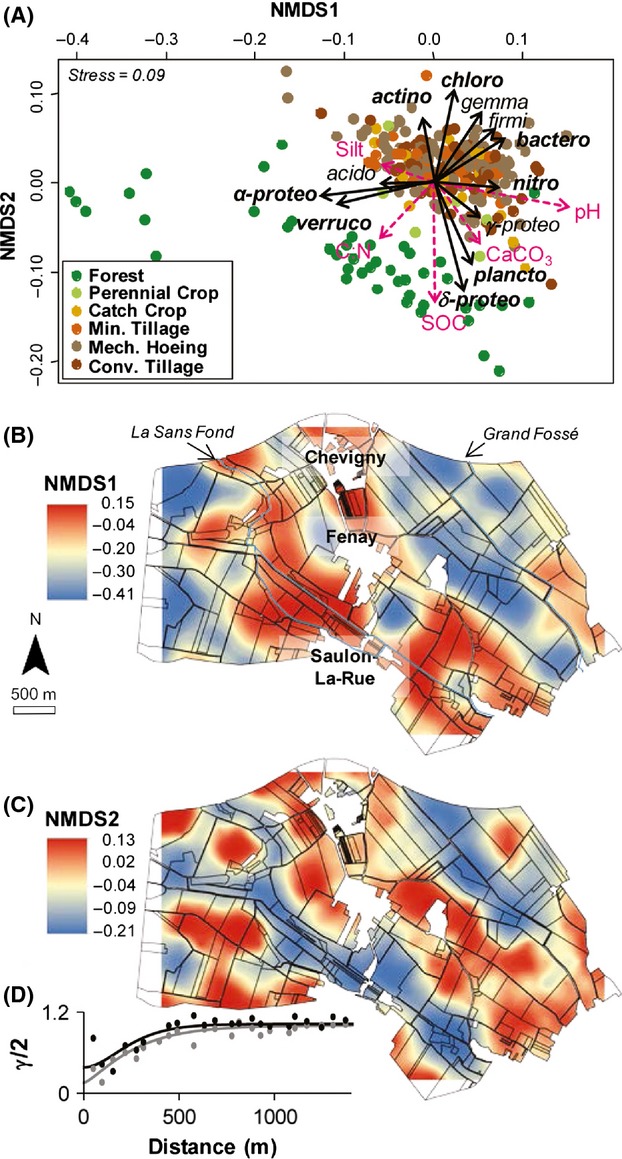
Nonmetric multidimensional scaling (NMDS) analysis derived from the Weighted Unifrac metric. (A) Ordination plot of the bacterial community structure. Vectors overlay were constructed based on the physicochemical properties (light red) and the relative abundance of discriminative phyla and *Proteobacteria* classes (black). Only significant correlations (≥0.20 with *P *<* *0.001)) are displayed. The angle and length of the vector indicate the direction and strength of the variable. Maps of the bacterial community structure based on the sample scores on NMDS first (B) and second dimension (C), thus, reflecting the community composition reduced to only two dimensions. The color scale to the left of each map indicates the extrapolated sample scores on the corresponding NMDS axis. (D) Semi-variograms of the transformed sample scores of NMDS1 (grey points and line for experimental and model variograms, respectively) and NMDS2 (black points and line, for experimental and model variograms, respectively).

Pyrosequencing of 16S rRNA genes yielded a total of 5 × 10^6^ sequences (10,800 quality sequences per sample), allowing taxonomic identification of the major bacterial and archaeal groups constituting the community in each soil sample. The NMDS ordination of Weighted UniFrac distance between samples revealed significant variation in community composition between soil samples across the landscape (Fig.2A). The NMDS stress of 0.09 confirmed that bacterial community could be accurately described in only two dimensions.

Vector fitting of the environmental parameters against the ordination plot of NMDS revealed that bacterial composition discrimination on the first axis was mainly explained by pH (*R*^2^: 0.82, *P *<* *0.001), and silt content (*R*^2^: 0.20, *P *<* *0.001), whereas the main environmental parameters involved in discrimination on the second axis were soil organic carbon (*R*^2^: 0.61, *P *<* *0.001), CaCO_3_ (*R*^2^: 0.36, *P *<* *0.001) and also silt contents (*R*^2^: 0.20, *P *<* *0.001) (Fig.2A). In addition, the bacterial communities under forests strongly differed from croplands on both axes in accordance with the lower pH, higher organic carbon content and higher C:N ratio (Fig.2A). No significant discrimination was observed in relation to the cropping intensity associated with the different agricultural land management clusters.

More precisely, the db-RDA analysis revealed that physicochemical data, land management and space explained 73% of the variation in community composition. This analysis confirmed that soil physicochemical characteristics and land management practices strongly contributed to community variation (24%, *P* < 0.001 and 7%, *P* *<* 0.001, respectively) and also revealed the significant marginal effect of space in shaping community variations (3%, *P* *<* 0.001).

Mapping of NMDS1 scores revealed a heterogeneous distribution of bacterial composition constituted by large patches with an effective range of 741 m (Fig.2B, Table S2). The bacterial community compositions were similar at the center of the studied area (i.e., all along the “*Sans-Fond”* riverbed and around the “*Chevigny*,” “*Fenay*” and “*Saulon-La-Rue*” local villages), and contrasted with the communities located at the extreme West and at the East (i.e., around the “*Grand Fossé*” riverbed) of the landscape (Fig.2B). The NMDS2 map exhibited smaller patches with a range of 574 m (Fig.2C, Table S2) and strong variations in community composition to the West and East of the studied area (Fig.2C). The robustness of these interpolated maps was supported by the cross validation statistics (Table S2).

The taxonomic affiliations at the phylum level, according to 16S rRNA gene sequences, revealed that the soils were generally dominated by *α*-Proteobacteria (mean relative abundance 23.6%, Table S1), *γ*-Proteobacteria (11.3%), Actinobacteria (11.2%), *δ*-Proteobacteria (10.8%), Bacteroidetes (8.4%), Acidobacteria (6.0%), and Firmicutes (5.5%). The bacterial and archaeal taxa involved in the bacterial community discrimination on the NMDS analysis were identified by vector fitting against the ordination plot. The main taxa explaining the community composition discrimination across this landscape were: on the first NMDS dimension, *α*-Proteobacteria (*R*^2^ = 0.78, *P* < 0.001), Verrucomicrobia (*R*^2^=0.49; *P* < 0.001), Nitrospirae (*R*^2^ = 0.26; *P* < 0.001) as well as *δ*-Proteobacteria (*R*^2^ = 0.76; *P* < 0.001), Chloroflexi (*R*^2^ = 0.53; *P* < 0.001), Bacteroidetes (*R*^2^ = 0.42; *P* < 0.001), Planctomycetes (*R*^2^ = 0.39; *P* < 0.001) on the second NMDS dimension. Forest samples were distinguished by a higher relative abundance of *α*-Proteobacteria, Verrucomicrobia, *δ*-Proteobacteria and Planctomycetes, and a lower relative abundance of Actinobacteria, Choloroflexi, Gemmatimonadetes, Firmicutes, Bacteroidetes, and Nitrospirae (Figs.2A and S2).

### Bacterial and archaeal phylum variation and mapping across landscape

**Figure 3:**
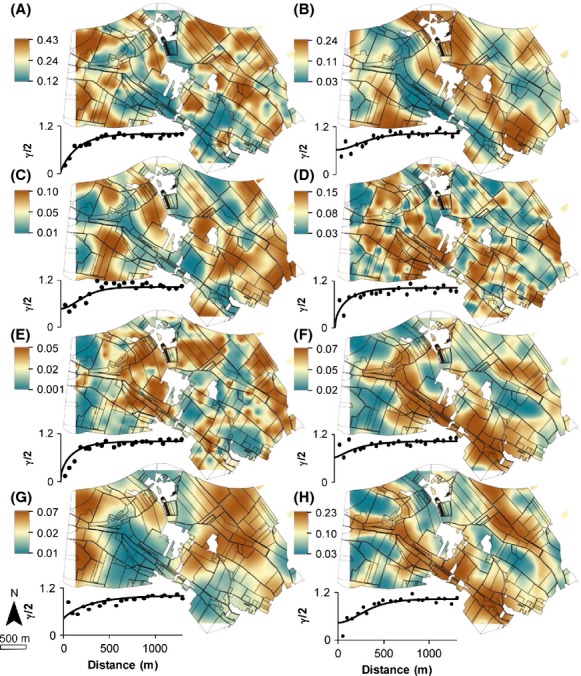
Maps of the relative abundance of most discriminative bacterial phyla and Proteobacteria classes across the Fénay landscape according to Figure2A. (A) *α*-proteobacteria; (B) Actinobacteria; (C) Chloroflexi; (D) Bacteroidetes; (E) Nitrospira; (F) Planctomycetes; (G) Verrucomicrobia and (H) d-proteobacteria. The color scale to the left of each map indicates the extrapolated relative abundance values. Semi-variograms used to describe and model the spatial pattern are provided beside each kriged map (experimental semi-variogram; points and models; lines).

The relative abundance of each bacterial phylum constituting the community on the krigged maps was interpolated by geostatistical approach. These maps evidenced a heterogeneous distribution of all the studied phylum, supported by the cross validation statistics (Table S2), with an effective patch range between 149 and 1147 m (Fig.3). As smaller spatial autocorrelation ranges were recorded for *γ*-*Proteobacteria* and *Acidobacteria* than in our usual sampling grid (i.e., <200 m, Table S2 vs. 215 m), no interpolated mapping was performed for these two bacterial taxa.

Four major patterns could be distinguished for phyla across the Fénay landscape and were ranked according to patch size. *α*-Proteobacteria, Bacteroidetes and Nitrospirae exhibited similar and “spotty” distributions, corroborated by autocorrelation ranges around 500 m, and low *v-*parameter values confirming raw spatial processes at smaller distances (Table S2). The maps of Fibrobacteres, Armatimonadetes, Gemmatimonadetes, Crenarchaeota, and *β*-proteobacteria across the landscape were patchier (Figs.3 and S1) with a spatial autocorrelation range around 600 m (Table S2). Planctomycetes, δ-Proteobacteria, Chloroflexi, Chlorobi, and Actinobacteria exhibited a spatial autocorrelation range around 700 m (Table S2), with high relative abundances for Planctomycetes, δ-Proteobacteria and Chlorobi, versus a lower relative abundance for Actinobacteria all along the “Sans Fond” riverbed (Figs.3B, F, H and S1). Finally, the distributions of Firmicutes, Thaumarchaeota, Verrucomicrobia, and Elusimicrobia were relatively smooth describing large patches (autocorrelation ranges around 1000 m, Table S2). More precisely, Firmicutes and Thaumarchaeota exhibited similar spatial distributions, which contrasted with the distribution of Verrucomicrobia (Figs.3 and S1).

### Variance partitioning of bacterial and archaeal taxa distribution

**Figure 4:**
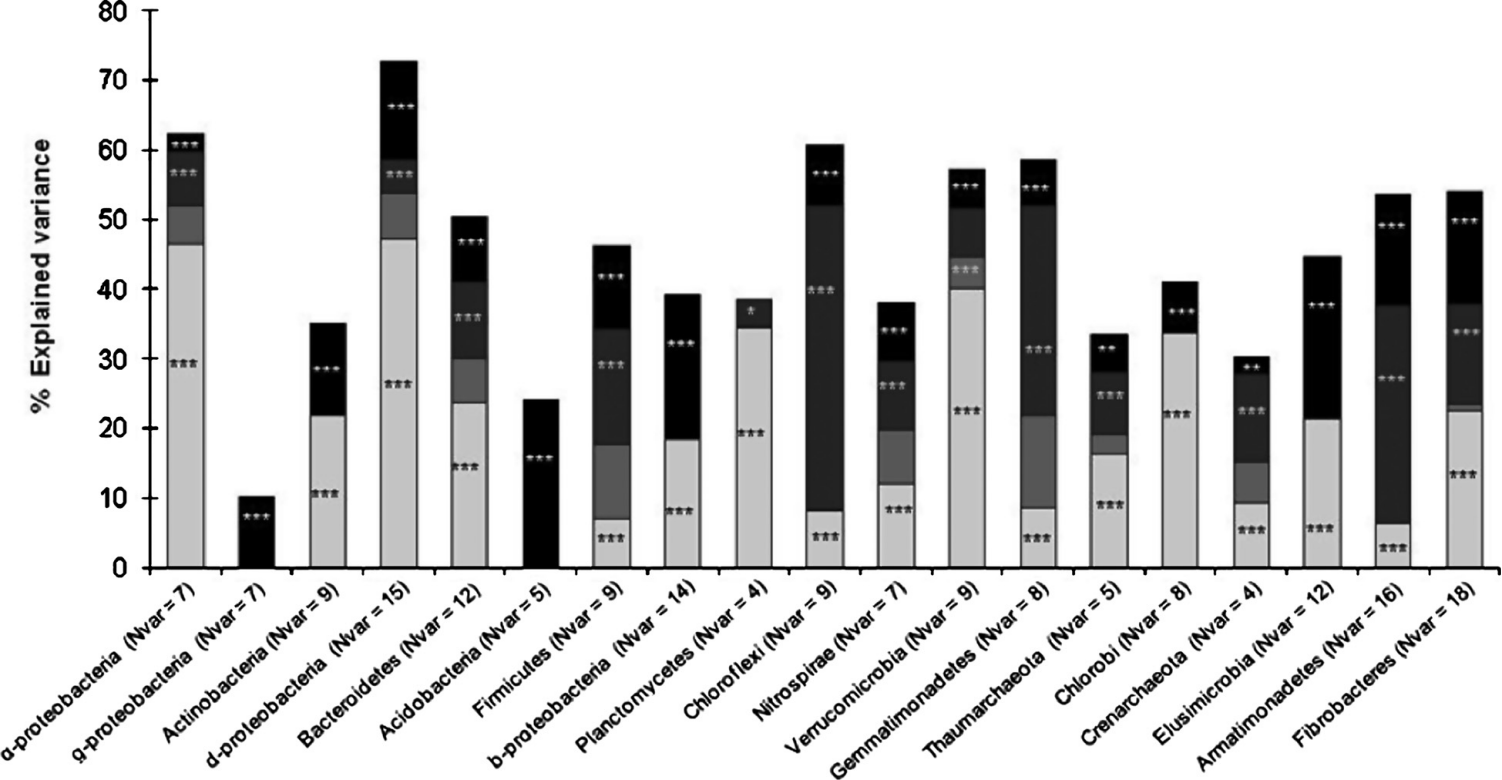
Partitioning of the variation of the bacterial phyla across the Fénay landscape according to environmental and spatial parameters. *N*_Var_ is the number of explanatory variables retained after selecting the most parsimonious explanatory variables (by minimizing the Akaike Information Criterion and maximizing the adjusted *R*^2^). Bacterial phyla and *Proteobacteria* classes are ranked from the most to the least abundant. The explained variance corresponds to the adjusted *R*^2^ values of the contextual groups of parameters (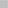: physicochemical characteristics, 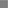: land management, 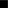: space and 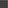: shared amount of variance between physicochemical properties and land management, using partial regressions). The significance level of the contribution of the sets of variables is indicated as follows; ns: not significant; *P* < 0.05: *; *P* < 0.01: **; *P* < 0.001: ***. Missing values indicate that no variable of the relating group was retained in the model.

A data set for soil physicochemical properties, land management and space was then used to partition the variance in taxa variation across the landscape. This approach demonstrated that between 10% and 73% of the total amount of variance could be explained according to taxa (Fig.4). The highest amount of explained variance was observed for *δ-*Proteobacteria, *α*-Proteobacteria, Chloroflexi, Gemmatimonadetes, and Verrucomicrobia (from 57% to 73%, Fig.4), whereas variations in *γ*-proteobacteria and Acidobacteria were weakly explained (10% and 24%, respectively, Fig.4).

Soil physicochemical parameters were the most important predictors for 10 out of the 19 phyla studied, and explained up to 47% of the total variance (Fig.4). On the other hand, Chloroflexi, Armatimonadetes, Gemmatimonadetes, and Firmicutes were mainly influenced by land management, which explained between 30% and 44% of their variation (Fig.4). Interestingly, the variations in a few bacterial phyla, including Actinobacteria, *β*-Proteobacteria, Chlorobi, and Elusimicrobia could not be significantly explained in terms of land management (Fig.4). Except for Planctomycetes, residual spatial autocorrelation was significantly involved in bacterial taxa variations and explained significant amounts of variance (from 2.4% to 24% Fig.4). Moreover, only spatial parameters were involved in explaining the variation of *γ*-Proteobacteria and Acidobacteria (10% and 24%, respectively, Fig.4).

**Figure 5:**
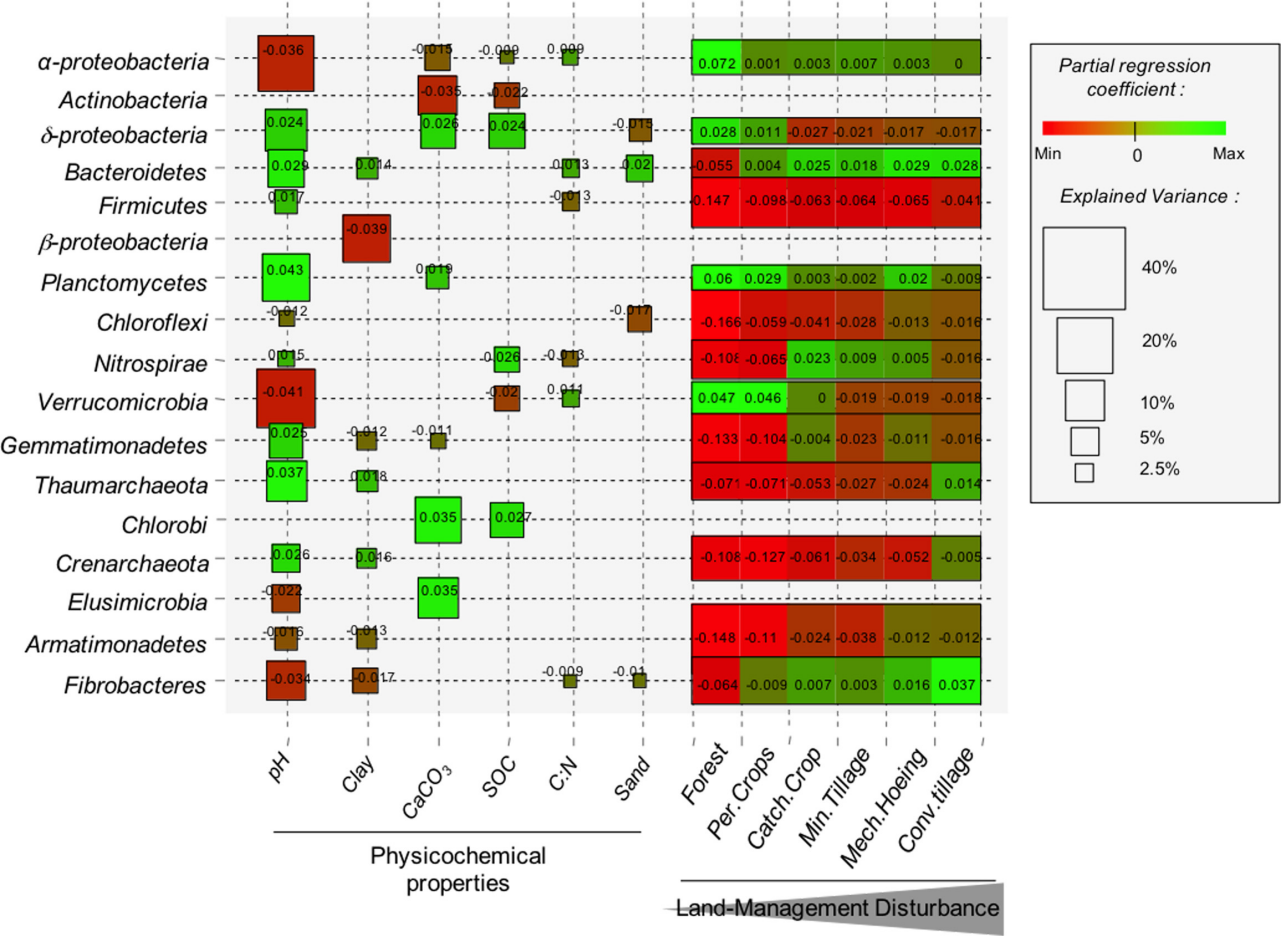
Contribution and effect of physicochemical and land management variables in the distribution of bacterial phyla. The respective significant contribution of each variable is represented by the height of the shape and was calculated by taking into account all other variables using partial regression models and adjusting the *R*^2^ values. The color was scaled to depict the value of the standardized partial regression coefficients (green, positive, red negative effect). *γ-Proteobacteria* and *Acidobacteria* are not represented since no significant contribution of any physicochemical or land management variables explained their variations in the data set. Bacterial taxa are ranked according to their overall relative abundance in the data set.

The marginal effects of each parameter within the sets of soil characteristics were ranked according to the respective amounts of variance explained, and to their standardized estimated coefficients, which indicated a positive or negative influence on bacterial and archaeal taxa variations. Only a small number of parameters were involved in explaining the distribution of phyla belonging to *β*-Proteobacteria, Chlorobi, and Firmicutes, (Fig.5), whereas a larger number of parameters were involved in determining the variation of phyla such as *α*-Proteobacteria, *δ*-Proteobacteria, Verrucomicrobia, and Fibrobacteres (Fig.5). Soil pH contributed in explaining the variation in 14 of the 19 studied phyla and explained the highest amounts of variance (Fig.5). More precisely, pH was positively correlated with the relative abundance of *δ*-Proteobacteria, Bacteroidetes, Planctomycetes, and Thaumarchaeota but negatively correlated with that of *α*-Proteobacteria, Verrucomicrobia, and Fibrobacteres (Fig.5). Clay and sand contents were involved in explaining variations in nine of the 19 studied phyla, but were only significant in explaining *β*-Proteobacteria variations through their negative influence on its relative abundance (Fig.5). CaCO_3_ content negatively impacted the variation of Actinobacteria, but positively affected that of *δ*-Proteobacteria, Chlorobi and Elusimicrobia (Fig.5). Soil organic carbon content and C:N ratio were involved in a small number of phyla variations and explained small amounts of these variations.

Land management was not included in the filter-ranking due to the impossibility of determining the relative contributions of each category. However, comparison of the signs and values of the standardized estimated coefficients highlighted the contrasting influences of land management intensity on taxa variation. A positive relationship was observed between cropping intensity (from forest to conventional tillage) and variations in bacterial and archaeal taxa belonging to Bacteroidetes, Firmicutes, Chloroflexi, Gemmatimonadetes, Thaumarchaeota, Crenarchaeota, and Fibrobacteres (Fig.5). On the contrary, a negative influence of cropping intensity was observed for *δ*-Proteobacteria, Planctomycetes, and Verrucomicrobia (Fig.5). An unusual response was observed for Nitrospira in that the response curve was hump-backed and centered on catch crop and minimum tillage (Fig.5).

## Discussion

Although spatial patterns of microbial diversity have been well documented from micro-scale (e.g., Constancias et al. 2014) to continental scale (e.g., Fierer and Jackson 2006) these studies did not provide significant insights into the processes and ecological attributes regulating bacterial composition and the populations constituting the whole community. Here, we focused on an agricultural landscape to determine the relative influence of land management intensity and soil physicochemical parameters on soil bacterial and archeal community composition and populations.

First, our study provided original maps of bacterial and archaeal community composition revealing significant spatial patterns and emphasizing that microbial communities are not randomly distributed at the landscape scale as previously observed at other scales (King et al. 2010). Visual comparison of the patches obtained for community composition and environmental characteristics revealed significant matches suggesting a significant influence of both land management and soil characteristics. This was statistically confirmed by the variance partitioning analysis, which also revealed that space explained a significant part of soil microbial community variation. This result implies that neither deterministic processes (environmental selection) nor neutral processes (dispersal limitation) are exclusive in explaining community composition variation (Martiny et al. 2011; Ranjard et al. 2013). A similar observation was reported for macroorganisms (Martiny et al. 2006), and for microorganisms at territorial or continental scales (Martiny et al. 2011; Ranjard et al. 2013) and more recently, it has been shown that models based on the neutral theory are able to predict the distribution patterns of microorganisms (Sloan et al. 2006; Woodcock et al. 2007).

Among the soil properties, pH was one of the most significant drivers of bacterial composition. Fierer and Jackson (2006) suggested that pH imposes significant and direct physiological stress on bacterial cells, selecting the best-adapted ones. The primary role of pH on bacterial community diversity and composition has been demonstrated in numerous studies over the past decade (e.g., Fierer and Jackson 2006; Rousk et al. 2010; Shen et al. 2013). In our case, even if pH exhibited weak variability (mean of 7.7, with a median of 8.0) across the landscape, it mainly influenced bacterial community variations. On the other hand, this small pH variation made it possible to show that texture, organic carbon content and C:N are also important drivers of bacterial community structure. Soil texture has been shown to control habitat number and diversity in terms of hosting and protecting microbial communities against several abiotic and biotic stresses, including desiccation and predation from protozoa, for example (Ranjard and Richaume 2001). Covariations between bacterial community composition and organic carbon content and C:N ratio confirmed the influence of nutrient quantity and quality on microbial community composition (Dequiedt et al. 2009). This covariation could result from competition between bacterial populations for different types of soil organic matter according to their copiotrophic/oligotrophic attributes (Bernard et al. 2007; Fierer et al. 2007).

Organic content, C:N ratio and pH are strongly impacted by land management and especially agricultural practices (Arrouays et al. 2001). This is coherent with the strong discrimination of bacterial communities observed between forest and croplands, which exhibited differences in soil characteristics. The influence of agricultural land management, which had been separated into different clusters based on cropping intensity and soil disturbance by tillage, did not reveal any significant discrimination of bacterial community composition between these clusters. This result is not in agreement with previous experimental trials where tillage intensity was shown to be an important driver of soil microbial communities (Acosta-Martínez et al. 2010; Lienhard et al. 2013). Therefore, soil characteristics rather than agricultural practices have a stronger influence on bacterial community composition at a landscape scale and only important modifications in land management type would impact bacterial community composition (Lauber et al. 2008; Kuramae et al. 2012).

By characterizing the distribution of bacterial and archaeal taxonomic groups at the landscape scale, we were able to compile, for the first time, original maps for the 19 most abundant phyla constituting the community. These maps revealed a heterogeneous and spatially structured distribution for all taxa except the Acidobacteria and *γ*-Proteobacteria phyla. The absence of significant spatial patterns for these two taxa might be partly due to the low pH variability across the landscape as pH is known to be an important driver for them (Lauber et al. 2008; Lauber et al. 2009; Nacke et al. 2011). It is interesting to note the contrasting distribution patterns of each bacterial taxon across the landscape, with patches ranging from 493 to 1147 m depending on the taxon. Different types of distribution patterns, characterized by large, medium or small patch size, could be distinguished at this scale. Bacterial taxa such as Bacteroidetes, Nitrospira, and *α*-Proteobacteria exhibited spatial patterns characterized by small patches (about 500 m), which partly matched with the distribution of land management types across the landscape. Fibrobacteres, Armatimonadetes, Gemmatimonadetes, Crenarchaeota, and *β*-Proteobaceria were distributed in larger patches (around 600 m), which matched both the distribution of land management clusters and physicochemical characteristics. Contrastingly, the spatial patterns of taxa including Chlorobi, Actinobacteria, Planctomycetes, and *δ*-Proteobacteria were characterized by an intermediate patch size (about 700 m) which matched with soil characteristics and geomorphology, especially in the case of the “Sans fond” river location. Finally, taxa belonging to Thaumarchaeota, Verrucomicrobia, Elusimicrobia, and Firmicutes exhibited larger patches across the landscape (about 1000 m), which also matched with variations in soil characteristics and geomorphology. These contrasting patterns suggest that different drivers contrastingly shape bacterial taxa across the landscape. It also suggests that bacterial taxa might be differentially influenced by neutral processes (i.e., dispersal capabilities).

Variance partitioning analysis of bacterial and archaeal taxa variation revealed that soil physicochemical characteristics and land management mainly contributed in explaining the spatial distribution of 16 of the 19 taxa. This suggests that the main process shaping the distribution of bacterial and archaeal taxa across the landscape is environmental selection determined by physicochemical properties and land-use. Repeated reports of the strong influence of local soil environmental heterogeneity had led to the conclusion that selection was the only process shaping soil microbial communities (Fierer and Jackson 2006; Rousk et al. 2010). Interestingly, we systematically recorded a significant contribution of space in explaining the distribution of bacterial and archaeal taxa (except for Planctomycetes), which suggests that dispersal may also contribute to producing the observed patterns. However, demonstrating the influence of a dispersal process in shaping the distribution of soil microbial communities and populations is neither easy nor frequent in microbial ecology with few studies using appropriate sampling designs and modeling approaches (Hanson and Fuhrman 2012). In our case, the contrasting contribution of space depending on the taxa could result from different dispersal capabilities, which would include their abilities for passive dispersal and to successfully settle in locations characterized by contrasting environmental conditions (Hanson and Fuhrman 2012). This differential contribution of space could also result from mass effects with populations being maintained at particular locations by the constant emigration of individuals from distant hotspots (Leibold et al. 2004). This could be especially important for phyla with spatial patches outside the range of soil physicochemical characteristics and land management practices (e.g., Nitrospirae, Bacteroidetes, Firmicutes and Elusimicrobia). On the other hand, the relatively poor impact of space in determining the distributions of bacterial and archaeal taxa belonging to *α*-Proteobacteria, Planctomycetes, Crenarchaeota, and Verrucomicrobia could reflect the weak impact of dispersal-mediated processes. This is in agreement with the size of the patch, which matches with physicochemical variability across the landscape.

Unsurprisingly, pH emerges as the filter exhibiting the most important correlation with the distribution of most of the phyla, thus confirming its strong influence on the community composition as a whole. *α*-Proteobacteria, *δ*-Proteobacteria, Planctomycetes, and Verrucomicrobia were strongly correlated with soil pH (both positively and negatively). The acidophilic attributes of some genera belonging to *α*-Proteobacteria and Verrucomicrobia and the basophilic attributes of some genera belonging to Planctomycetes and *δ*-Proteobacteria are coherent with the correlation between these taxa and pH reported in recent studies (Nacke et al. 2011). Soil texture, represented by clay or sand contents, was the second most important soil driver for *β*-Proteobacteria, Bacteroidetes, and Chloroflexi. This suggests that some taxa are better adapted to live in less protected and oligotrophic habitats represented by coarse textured soils whereas others live in more protected and copiotrophic habitats represented by fine textured soils (Dequiedt et al. 2009; Constancias et al. 2014). More precisely, *β*-Proteobacteria were negatively influenced by clay content indicating that coarse textured soils are more favorable habitats for this taxon. These observations confirmed the affinity of some genera belonging to *β*-proteobacteria and Bacteroidetes for a disturbed environment and matched with their ecological attributes as r-strategists (Cleveland et al. 2007). Soil organic carbon content and C:N ratio, representing trophic quantity and quality, were less shared drivers of the bacterial and archaeal taxa and explained smaller amounts of their variation. This contrasts with Fierer et al. (2007), who demonstrated experimentally that most of the bacterial phyla could be simply described according to their copiotrophic and oligotrophic attributes. This discrepancy could result from the low variations in soil organic content and C:N ratio that occurred across the studied landscape. Nevertheless, the spatial distribution of *δ*-Proteobacteria, Chlorobi and Actinobacteria was mainly influenced by soil organic content. More precisely, *δ*-Proteobacteria and Chlorobi were positively influenced whereas Actinobacteria was negatively affected by trophic quantity, thus confirming the respective copiotrophic and oligotrophic behaviors of some genera belonging to these phyla (Cleveland et al. 2007; Pascault et al. 2013).

The confrontation of soil bacterial and archaeal taxa variation with land use revealed that *α-*Proteobacteria, Fibrobacteres, and Bacteroidetes phyla were strongly impacted by a coarse level of land use discrimination (forest vs. croplands). These observations confirmed recent studies which highlighted a greater relative abundance of Fibrobacteres, Bacteroidetes, and a lower relative abundance of *α-*Proteobacteria in agricultural soils as compared to forest ecosystems (Jangid et al. 2008; Nacke et al. 2011; Shange et al. 2012). Similarly, the distributions of *δ-*Proteobacteria, Planctomycetes, Verrucomicroba, and Gemmatimonadetes were impacted by an increasing cropping intensity represented by crops versus forest and perennial crops. Planctomycetes and *δ*-Proteobacteria, which have been described as *K*-strategists, (Buckley et al. 2006; Pascault et al. 2013) might have an advantage under less disturbed environmental conditions. In the Fénay landscape, the catch crop mainly consisted in leguminous plants that could explain the observed ecological optimum of Nitrospirae*,* which includes taxa known to interact with plant communities. Bacterial and archaeal taxa including Bacteroidetes, Thaumarchaeota, Crenarchaeota, Armatimonadetes, and Fibrobacteres exhibited their ecological optima at the highest level of land management disturbance, represented by conventional tillage. Bacteria belonging to the Bacteroidetes phyla have been recently described as r-strategists and stress resistant which could explain their affinity for highly disturbed soil environments (Eilers et al. 2010). However, Thaumarchaeota, Crenarchaeota, Armatimonadetes, and Fibrobacteres are usually pooled as minor taxa (<1%), and therefore, to date, we do not possess any significant knowledge about their ecological attributes. Nevertheless, our study suggests that they can be considered as *r*-strategists.

Altogether, by studying bacterial community composition and taxa distribution at a landscape scale, we evidenced that the distribution of each taxon, as well as the community composition as a whole, is heterogeneous and spatially structured. The results of our study also emphasize that environmental selection may not be the only process that explains patterns of soil microbial community distribution. The selection process results from soil physicochemical filters (pH, texture and nutrient status), to a large extent, but also from disturbance intensity arising from human activities. Even though our study did not directly demonstrate that the influence of space was exclusively due to dispersal limitation of the populations constituting the community, our data would support this hypothesis. In addition, a spatial approach was used to complete and define new ecological attributes for most of the taxa identified. Further investigations should now be devoted to the spatial patterns of fungal communities to fully depict the mechanisms and drivers of soil microbial biodiversity, and a more thorough analysis of the link with soil functioning.

## Supporting Information

**Table S1.**Summary statistics of sample characteristics (*n* = 278). **Table S2.** Geostatistics and cross-validation parameters of the physicochemical properties, bacterial community composition and bacterial population. **Figure S1.** Additional maps to Figure 3 displaying the relative abundance of bacterial *phyla* and *Proteobacteria* classes across the Fénay landscape. The color scale to the left of each map indicates the extrapolated relative abundance values.
