# Supplementary material for: Contrasting spatial patterns and ecological attributes of soil bacterial and archaeal taxa across a landscape
Source: Microbiologyopen. 2015 Apr 28;4(3):518–31. doi: 10.1002/mbo3.256 (PMC4475392; doi:10.1002/mbo3.256)
Supplement: Supplementary file 1 — Table S1.Summary statistics of sample characteristics (n = 278). Table S2. Geostatistics and cross-validation parameters of the physicochemical properties, bacterial community composition and bacterial population. Figure S1. Additional maps to Figure 3 displaying the relative abundance of bacterial phyla and Proteobacteria classes across the Fénay landscape. The color scale to the left of each map indicates the extrapolated relative abundance values. [file mbo30004-0518-sd1.docx]

**Supplementary information:**

**Table S1: Summary statistics of sample characteristics (n=278).**

|  | Mean (SD) | | Median | [min; max] |
| --- | --- | --- | --- | --- |
| **Physicochemical** | | |  |  |
| Organic Carbon (g.kg^-1^) | 21.9 (15.8) | | 17.1 | [1.7; 174] |
| total Nitrogen (g.kg^-1^) | 2 (1.3) | | 1.6 | [0.8; 14.6] |
| C:N ratio | 10.7 (1.6) | | 10.4 | [1; 22.2] |
| pH | 7.7 (0.7) | | 8.0 | [4.7; 8.4] |
| CaCO_3_ (g.kg^-1^) | 84.6 (161.2) | | 3.3 | [0; 835] |
| Clay (%) | 33.3 (9.5) | | 34.3 | [8; 61.7] |
| Silt (%) | 57.9 (9.6) | | 56.7 | [35.5; 86.2] |
| Sand (%) | 8.8 (4.8) | | 7.4 | [2; 29.3] |
| **Bacterial populations** (relative abundance) | | |  |  |
| *α-Proteobacteria* | | 0.236 (0.04) | 0.235 | [0.12; 0.43] |
| γ-*Proteobacteria* | | 0.113 (0.02) | 0.111 | [0.07; 0.34] |
| *Actinobacteria* | | 0.112 (0.03) | 0.111 | [0.03; 0.24] |
| *δ-Proteobacteria* | | 0.108 (0.03) | 0.101 | [0.03; 0.24] |
| *Bacteroidetes* | | 0.084 (0.02) | 0.085 | [0.04; 0.15] |
| *Acidobacteria* | | 0.06 (0.02) | 0.058 | [0.04; 0.22] |
| *Firmicutes* | | 0.055 (0.01) | 0.055 | [0.03; 0.1] |
| *β-Proteobacteria* | | 0.053 (0.01) | 0.051 | [0.03; 0.1] |
| *Planctomycetes* | | 0.05 (0.01) | 0.049 | [0.02; 0.07] |
| *Chloroflexi* | | 0.049 (0.02) | 0.049 | [0.01; 0.1] |
| *Nitrospirae* | | 0.022 (0.01) | 0.022 | [0; 0.05] |
| *Verrucomicrobia* | | 0.018 (0.01) | 0.016 | [0.01; 0.07] |
| *Gemmatimonadetes* | | 0.01 (0) | 0.010 | [0; 0.02] |
| *Thaumarchaeota* | | 0.009 (0.01) | 0.007 | [0; 0.05] |
| *Chlorobi* | | 0.007 (0.01) | 0.005 | [0; 0.03] |
| *Crenarchaeota* | | 0.004 (0) | 0.003 | [0; 0.02] |
| *Elusimicrobia* | | 0.002 (0) | 0.002 | [0; 0.01] |
| *Armatimonadetes* | | 0.002 (0) | 0.002 | [0; 0.01] |
| *Fibrobacteres* | | 0.001 (0) | 0.000 | [0; 0.01] |

**Table S2: Geostatistics and cross-validation parameters of the physicochemical properties, bacterial community composition and bacterial population.**

|  | Nugget^a^ | Sill^b^ | Effective Range^c^ | Structural Variance^d^ | ν parameter^e^ | µ(**θ)**^f^ | median**(θ)**^f^ |
| --- | --- | --- | --- | --- | --- | --- | --- |
| **Physicochemical characteristics** | | | | | | | |
| PC Axis 1 | 0.07 | 1.01 | 622 | 93% | 2.0 | **1.00** | **0.38** |
| PC Axis 2 | 0.31 | 1.04 | 528 | 70% | 2.5 | **1.02** | **0.46** |
| PC Axis 3 | 0.00 | 0.99 | 919 | 100% | 0.3 | **1.00** | **0.38** |
| **Bacterial community structure** | | | |  |  |  |  |
| NMDS 1 | 0.17 | 1.02 | 741 | 83% | 0.8 | **1.00** | **0.57** |
| NMDS 2 | 0.39 | 1.03 | 574 | 62% | 3.0 | **1.00** | **0.34** |
| **Bacterial populations** (relative abundance) | | | | | | | |
| α-*Proteobacteria* | 0.04 | 1.02 | 573 | 96% | 0.5 | **1.01** | **0.53** |
| *Actinobacteria* | 0.63 | 1.04 | 744 | 40% | 3.0 | **1.00** | **0.49** |
| *Chloroflexi* | 0.47 | 1.02 | 597 | 54% | 3.0 | **1.00** | **0.41** |
| *Bacteroidetes* | 0.00 | 1.01 | 457 | 100% | 0.3 | **1.00** | **0.45** |
| *Nitrospira* | 0.00 | 1.01 | 448 | 100% | 0.3 | **1.01** | **0.46** |
| *Planctomycetes* | 0.62 | 1.02 | 719 | 39% | 0.8 | **1.00** | **0.46** |
| *Verrucomicrobia* | 0.43 | 1.01 | 974 | 57% | 0.4 | **1.00** | **0.51** |
| δ-*Proteobacteria* | 0.45 | 1.04 | 754 | 57% | 3.0 | **0.99** | **0.45** |
| *γ-Proteobacteria* | 0.00 | 1.01 | *190* | 100% | 0.30 | **1.00** | **0.48** |
| *Acidobacteria* | 0.00 | 1.00 | *149* | 100% | 3.00 | **0.99** | **0.47** |
| *Firmicutes* | 0.49 | 1.06 | 1147 | 54% | 0.62 | **1.01** | **0.42** |
| *β-Proteobacteria* | 0.44 | 1.02 | 623 | 57% | 1.20 | **1.00** | **0.42** |
| *Gemmatimonadetes* | 0.52 | 1.01 | 672 | 49% | 0.66 | **1.00** | **0.57** |
| *Thaumarchaeota* | 0.54 | 1.07 | 843 | 50% | 3.00 | **1.00** | **0.41** |
| *Chlorobi* | 0.45 | 1.04 | 562 | 57% | 3.00 | **1.00** | **0.35** |
| *Crenarchaeota* | 0.52 | 1.03 | 762 | 49% | 0.89 | **1.00** | **0.43** |
| *Elusimicrobia* | 0.58 | 1.03 | 1044 | 44% | 0.49 | **1.00** | **0.46** |
| *Armatimonadetes* | 0.45 | 1.02 | 493 | 56% | 3.00 | **1.01** | **0.57** |
| *Fibrobacteres* | 0.48 | 1.01 | 512 | 52% | 3.00 | **0.99** | **0.46** |

Data were standardized by Gaussian quantile transformation.

^a)^ Nugget variance (C_o_), the magnitude of the discontinuity of the variogram.

^b)^ Sill (C_o_+C_1)_, the value of the variogram for distances beyond the range of the variogram.

^c)^ Effective range (m)

^d)^ Structural variance (%), calculated as (C_1_/(C_o_+C_1_) which represents the amount of variance spatially structured.

^e)^ Kappa, the Matérn smooth parameter

^f)^ Mean and median of the cross validation statistic (**θ**), bold values are in the 0.95 confidence interval.


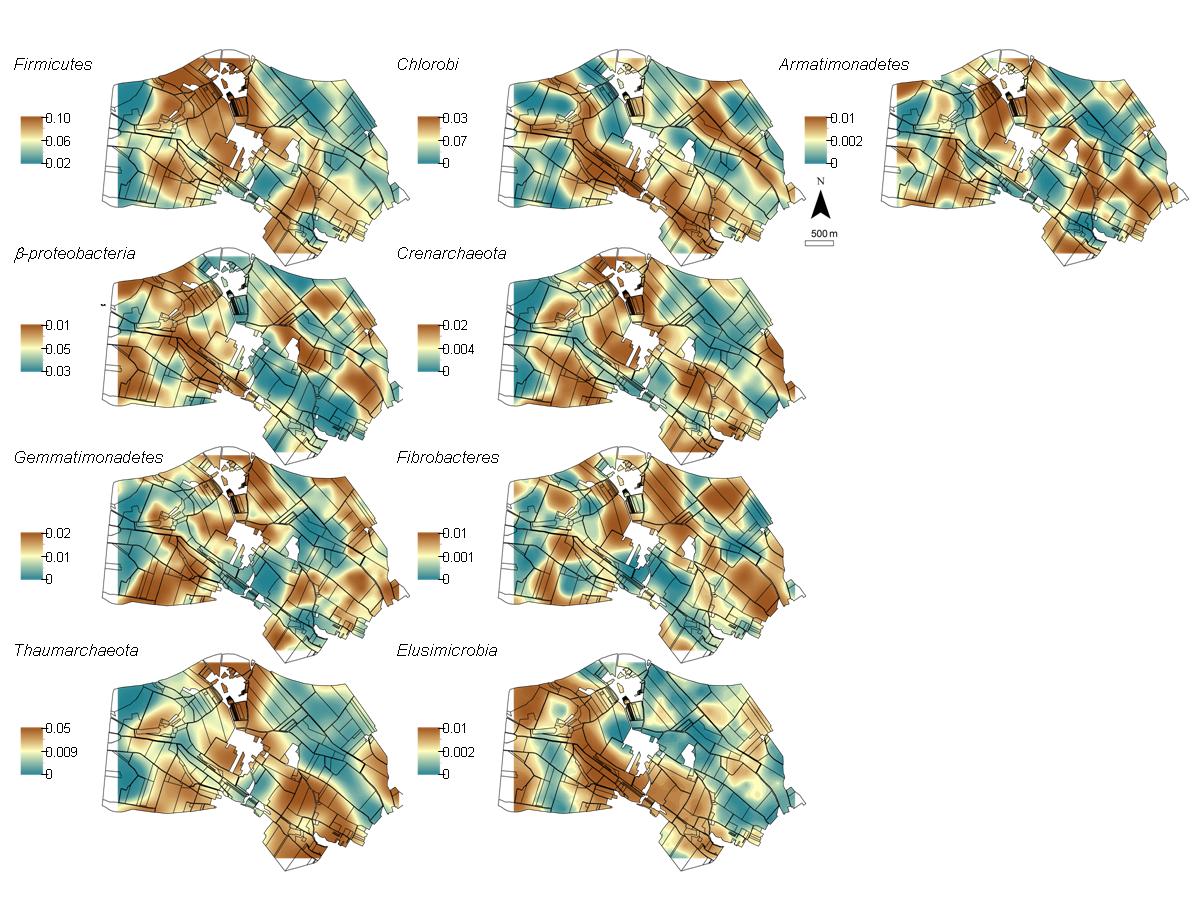


**Figure S1:** Additional maps to Figure 3 displaying the relative abundance of bacterial *phyla* and *Proteobacteria* classes across the Fénay landscape. The colour scale to the left of each map indicates the extrapolated relative abundance values
